# Klotho and SIRT1 changes from pre-diabetes to diabetes and pre-hypertension to hypertension

**DOI:** 10.1186/s13098-021-00736-2

**Published:** 2021-10-20

**Authors:** Mahboobeh Yeganeh-Hajahmadi, Hamid Najafipour, Farzaneh Rostamzadeh, Ahmad Naghibzadeh-Tahami

**Affiliations:** 1 Physiology Research Center, Institute of Neuropharmacology, Kerman University of Medical Sciences, Kerman, Iran; 2 Cardiovascular Research Center, Institute of Basic and Clinical Physiology Sciences, Kerman University of Medical Science, Kerman, Iran; 3 Endocrinology and Metabolism Research Center, Institute of Basic and Clinical Physiology Sciences, Kerman University of Medical Sciences, Jehad Blvd, Ebn Sina Avenue, 76198-13159 Kerman, Iran

**Keywords:** Klotho, Sirt1, Pre-hypertension, Hypertension, Pre-diabetes, Diabetes mellitus

## Abstract

**Background:**

Hypertension and diabetes are among the most important risk factors of cardiovascular diseases. Klotho and SIRT1 are known as anti-aging factors with beneficial effects on cardiovascular system. In this study we investigated the serum Klotho and SIRT1 levels in pre-diabetic and pre-hypertensive individuals and then in diabetic and hypertensive patients to see their relationship with these diseases.

**Method:**

229 individuals divided into six groups with similar gender and age distribution 1—Control (normal BP and FBS) 2—pre-diabetic (FBS between 100 and 125 mg/dl) 3—diabetic (FBS ≥ 126 mg/dl), 4—pre-hypertensive (SBP 120–139 or DBP 80–89 mm Hg) 5—hypertensive (SBP ≥ 140 or DBP ≥ 90 mm Hg), and 6—patients with combined hypertension/diabetes. Serum levels of Klotho and SIRT1 were measured by ELISA method.

**Results:**

Serum Klotho and STRT1 levels decreased in pre-diabetes and returned to normal in diabetic patients. Their concentration increased in pre-hypertension and recovered to normal in hypertension. In the physiologic range of FBS there is a negative correlation between Klotho and SIRT1 with FBS. When pathologic ranges of FBS added to analysis, the negative correlation abolished/U shaped. Also an inverse U shape correlation observed between Klotho and SIRT1 with MAP in the range of normal to hypertensive BP levels. There was an overall positive relationship between the serum levels of Klotho and SIRT1 themselves.

**Conclusion:**

The serum levels of the anti-aging proteins Klotho and SIRT1 increases or reduces at the onset of the disease, as a compensatory mechanism, but as the disease progresses their level recovers.

## Introduction

Hypertension is a complex disorder caused by various genetic, environmental, and social factors [1]. The prevalence of hypertension is about 45.6% in American adults [2]. Hypertension is a risk factor for coronary artery disease, stroke, disability and death, and it is responsible for nearly ten million deaths worldwide annually [3].

The global prevalence of diabetes has increased dramatically in the last 3 decades, from 30 million in 1985 to 415 million in 2017 [4]. In the United States, diabetes is the leading cause of end stage renal disease (ESRD), lower limb amputation, and blindness in adults. It is also a predisposing factor for cardiovascular disease. As its global prevalence increases, diabetes is likely to be the leading cause of future disability and mortality [4]. Therefore, it is important to study the underlying mechanisms of hypertension and diabetes.

The prevalence of hypertension and related cardiovascular diseases as well as diabetes increases with age [4, 5], and in fact these diseases are age-dependent. Klotho is an anti-aging protein that is encoded by Klotho gene in the form of a membrane protein with an extracellular domain [6]. The circulating form of Klotho, which is detectable in plasma and urine, is known as soluble Klotho and is formed by the separation of the extracellular domain of the membrane-bound Klotho [6]. Klotho’s genetic defect leads to premature aging phenotype and drastically reduces life expectancy [7], while increasing its gene expression increases the life expectancy [8]. The early vascular aging which is seen in hypertension, diabetes and renal failure, is associated with decreased renal expression and decreased levels of Klotho in serum and urine [9]. Klotho induces its effects even in tissues which do not express it, indicating its endocrine role [6].

Sirtuins are a family of enzymes that have been shown to play an important role in longevity and health. There are seven sirtuins in mammals which are encoded by SIRT1–SIRT7. These enzymes are abundantly expressed in the nucleus and cytoplasm of several tissues, including the heart and vascular endothelium [10]. SIRT1 is the most well-known member of this family and has been shown to play a beneficial role in age associated metabolic, inflammatory and cardiovascular diseases. There is strong evidences that SIRT1 has anti-inflammatory, anti-oxidant, and anti-apoptotic effects in endothelium and thus prevents endothelial aging and dysfunction [11, 12]. After the first report showing that SIRT1 activates eNOS [13], several studies using mutated rats showed that SIRT1 has protective effects against atherosclerosis [11–14]. It has been shown that SIRT1 activation has beneficiary effects on aging and metabolic disorders such as metabolic syndrome and diabetes mellitus, and on chronic inflammatory diseases such as arthritis and atherosclerosis, and also on DNA damage and oxidative stress [15].

A recent study in rats with Klotho haplo-deficiency showed that this genetic defect is associated with arterial stiffening and hypertension and with decreased expression of SIRT1; and activating the SIRT1 gene abolished these defects [16]. Therefore, the primary aim of this study was to evaluate the serum levels of Klotho and SIRT1 in patients with high blood pressure and diabetes and the patients who have both diseases simultaneously. In this regard their serum level in pre-diabetes and pre-hypertension conditions are also assessed to consider if these the changes in the level of two proteins may be introduced as biomarker of the progression of these diseases. The secondary aim of the study was to investigate the correlation between the serum levels of these two proteins.

## Materials and methods

### Participants

The study subjects were selected from the phase 2 of a cohort study named KERCADRS (The Kerman Coronary Artery Disease Risk Study). The KERCADRS was performed on 10,000 individuals aged 15–80 years in Kerman, a city in south east of Iran, between 2014 and 2018 [17]. The study sampling method was a random one-stage cluster sampling. People invited to the study based on random selection of 420 zip codes among city zip codes and referring to their houses to invite them. All participants signed a written informed consent. They were physically examined by a physician [taking blood pressure (BP), past medical history, asking for coronary artery disease risk behaviors and current medicines], and interviewed for their demographic data and measuring height and weight. BP was evaluated after at least 10 min at rest and in sitting position. If abnormal, it was measured again at least 30 min after the first measurement at the same conditions. A 10–12 h fasting blood sample was taken for laboratory tests (serum lipids, glucose and HbA1C) at the time of attendance to the study site. The sera were kept in a – 70 °C freezer until the day of measurement. Details on how to measure biochemical factors and keep the lab samples are given in an article about the research methodology [18].

### Studied groups

The present study is a subgroup selection of 229 individuals among those 10,000 people, based on the aim of the study, and are divided in six groups: 1—control group (normal BP/normal FBS) 2—pre-diabetic individuals 3—diabetic individuals 4—pre-hypertensive individuals 5—hypertensive patients and 6—patients with combined hypertension and diabetes. The sample size was selected based on a previous study in which serum SIRT1 levels was measured [19]. There was 40 people in each group except for the sixth group in which there was 26 individuals (Given that we excluded patients who received treatment for their diseases, we couldn’t find more subjects with both hypertension and diabetes). Diabetic and pre-diabetic individuals were selected based on the guideline of the American diabetes association: for the present study every individual who had FPG  ≥  126 mg/dl at the time of recruitment (provided that the HbA1c is more than 6.5%) was considered as diabetic. Those with FPG between 100 and 125 mg/dl were considered pre-diabetic. Participants with FPG ≥ 126 mg/dl explored for the first time were recalled and HbA1c was checked, and if HbA1c was more than 6.5% confirmed the presence of diabetes. Pre-hypertensive people had systolic blood pressure (SBP) between 120 and 139 mm Hg and/or diastolic blood pressure (DBP) between 80 and 89 mm Hg. Hypertensive people had SBP ≥ 140 mm Hg and/or DBP ≥ 90 mm Hg [4]. All patients taking anti-diabetic or anti-hypertensive drugs, those with obesity and abnormal blood lipids were excluded from the study to avoid the probable adverse effects of medication or other metabolic disorders on the serum level of Klotho and SIRT1. Mean arterial pressure (MAP) was calculated by the formula: MAP = DBP + 1/3(SPB-DBP). Control group were participants who had normal blood pressure, blood sugar and lipids. The groups were similar in terms of gender and age distribution (see Table 1).

**Table 1:** Clinical and demographic characteristics of individuals in different study groups

	CTL	Pre-DM	DM	Pre-HTN	HTN	DM + HTN
Number	40	41	40	41	41	26
Sex (male)	20 (50%)	20 (48.8%)	16 (40%)	21 (51.2%)	20 (48.8%)	13 (50%)
Age (years)	58.05 ± 10.7	58.9 ± 10.5	58.7 ± 11.8	58.4 ± 10.2	58.6 ± 10.3	58.5 ± 10.4
BMI (Kg/m^2^)	25.7 ± 3.3	25.9 ± 2.9	25.1 ± 3.5	24.9 ± 3.2	25.7 ± 3.1	26.8 ± 2.5
FBS (mg/dl)	86.2 ± 7.6	108.7 ± 7.2*	166.7 ± 48.3*	86.5 ± 7.0	85.9 ± 6.7	170.6 ± 45.32*
HbA1C (%)	NM	NM	7.46 ± 2.5	NM	NM	7.5 ± 2.1
SBP (mmHg)	106.5 ± 6.3	116.7 ± 13.6*	101.3 ± 9.9	129.7 ± 4.6*	153.9 ± 16.7*	141.3 ± 14.7*
DBP (mmHg)	65.5 ± 6.5	72.7 ± 5.7*	67.0 ± 6.1	75.0 ± 6.7*	95.9 ± 11.0*	87.9 ± 7.8*
MAP (mmHg)	79.2 ± 5.7	87.4 ± 5.3	78.4 ± 6.7	93.3 ± 5.3	115.3 ± 11.0*	105.7 ± 7.1*
Cholesterol (mg/dl)	185.2 ± 30.4	185.3 ± 33.6	186.4 ± 31.8	187.0 ± 30.3	192.3 ± 30.8	187.5 ± 34.2
Triglyceride(mg/dl)	119.2 ± 89.7	143.6 ± 88.0	146.8 ± 101.7	111.0 ± 61.4	140.4 ± 72.4	131.9 ± 65.9
HDL (mg/dl)	42.9 ± 11.7	43.0 ± 11.2	47.1 ± 11.3	46.9 ± 11.8	49.9 ± 11.9	51.9 ± 11.6
LDL (mg/dl)	119.7 ± 23.3	113.7 ± 31.4	111.7 ± 30.7	117.9 ± 29.1	115.1 ± 25.0	114.2 ± 26.3

Data are expressed as mean  ±  SD or median (interquartile range)

*NM* not measured; *CTL* control; *Pre-DM* pre-diabetes; *DM* diabetes mellitus; *Pre-HTN* pre-hypertension; *HTN* hypertension

^*^P  <  0.01 vs control

### Klotho and SIRT1 measurement

Serum levels of Klotho and SIRT1 were measured by ELISA method using Human Klotho ELISA kit (R&D Systems with coefficient of variation < 10%) and Human SIRT1 ELISA kit (Elab Science with coefficient of variation < 10%).

### Statistical analysis

Statistical analysis was performed using SPSS 20. Normal distribution of data was tested with Kolmogorov–Smirnov test. Values are expressed as mean ± standard deviation for normally distributed variables (Age, FBS, BMI, SBP, DBP, MAP, HbA1C, Cholesterol, Triglyceride, HDL, LDL), and as median (interquartile range) for non-normally distributed variables (serum Klotho and SIRT1 levels). For comparison of two means of independent samples, t test or Mann–Whitney U test was used. For comparison of more than two means of independent samples, ANOVA test was used, followed by Tukey post-hoc analysis. To investigate the relation between serum Klotho and SIRT1 levels, linear regression was used. Multiple linear regression analysis was used to determine the correlation between serum Klotho and SIRT1 levels with FBS and MAP. All P values reported were two-tailed, and P values of  <  0.05 were considered as statistically significant.

## Results

### Clinical and demographic characteristics

Clinical and demographic characteristics of individuals in the studied groups are shown in Table 1. Except for the level of blood pressure and FBS, no significant differences was between the studied groups regarding demographic and other clinical characteristics, showing the groups are matched regarding other biochemical variables.

### Serum Klotho and SIRT1 levels

**Fig. 1:**
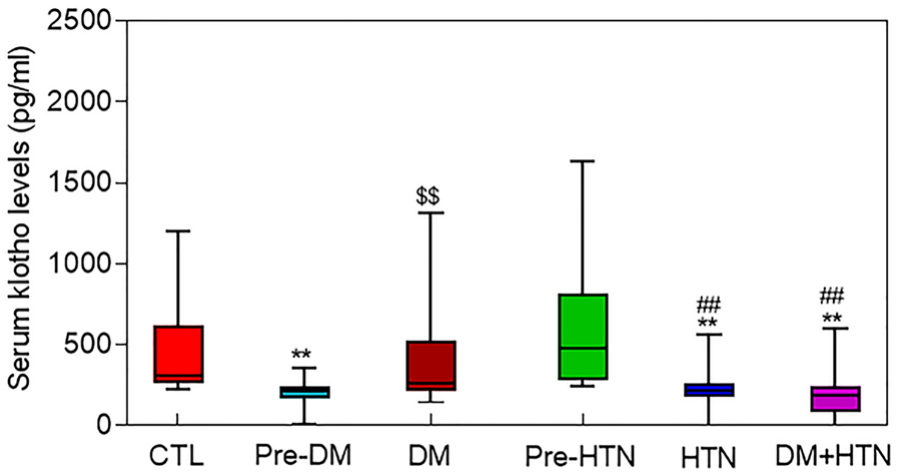
Box and whisker plot of serum Klotho levels among different groups. The boxes represent the 25th and 75th percentiles, and the central lines in the boxes represent the median values. Whiskers are presented as median ± 95% CI. *CTL* control; *Pre-DM* pre-diabetes; *DM* diabetes mellitus; *Pre-HTN* pre-hypertension; *HTN* hypertension. **P  <  0.01 vs. CTL, ^$$^P  <  0.01 vs. Pre-DM, ^##^P  <  0.01 vs. Pre-HTN

Serum Klotho level was significantly lower in people with pre-DM compared to the control and DM groups, while in DM patients the serum level recovered to control level. In people with HTN, and in those who had DM + HTN, serum Klotho level was significantly lower than in control group. Also, HTN group had lower Klotho levels than the pre-HTN group (Fig. 1).

**Fig. 2:**
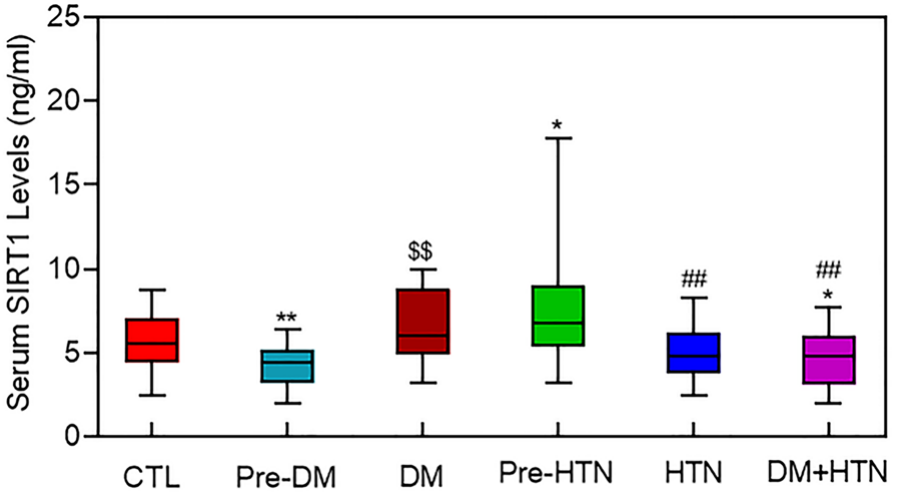
Box and whisker plot of serum Sirt1 levels among different groups. The boxes represent the 25th and 75th percentiles, and the central lines in the boxes represent the median values. Whiskers are presented as median ± 95% (CI). *CTL* control; *Pre-DM* pre-diabetes; *DM* diabetes mellitus; *Pre-HTN* pre-hypertension; *HTN* hypertension. *P  <  0.05 vs. CTL, **P  <  0.01 vs. CTL, ^$$^P  <  0.01 vs. Pre-DM, ^##^P  <  0.01 vs. Pre-HTN

Changes in serum SIRT1 levels was almost similar to the pattern of changes in serum Klotho, except that SIRT1 was significantly higher in pre-hypertensive individuals compared to the control group (Fig. 2).

### Klotho and SIRT1 correlation with FBS and MAP

**Fig. 3:**
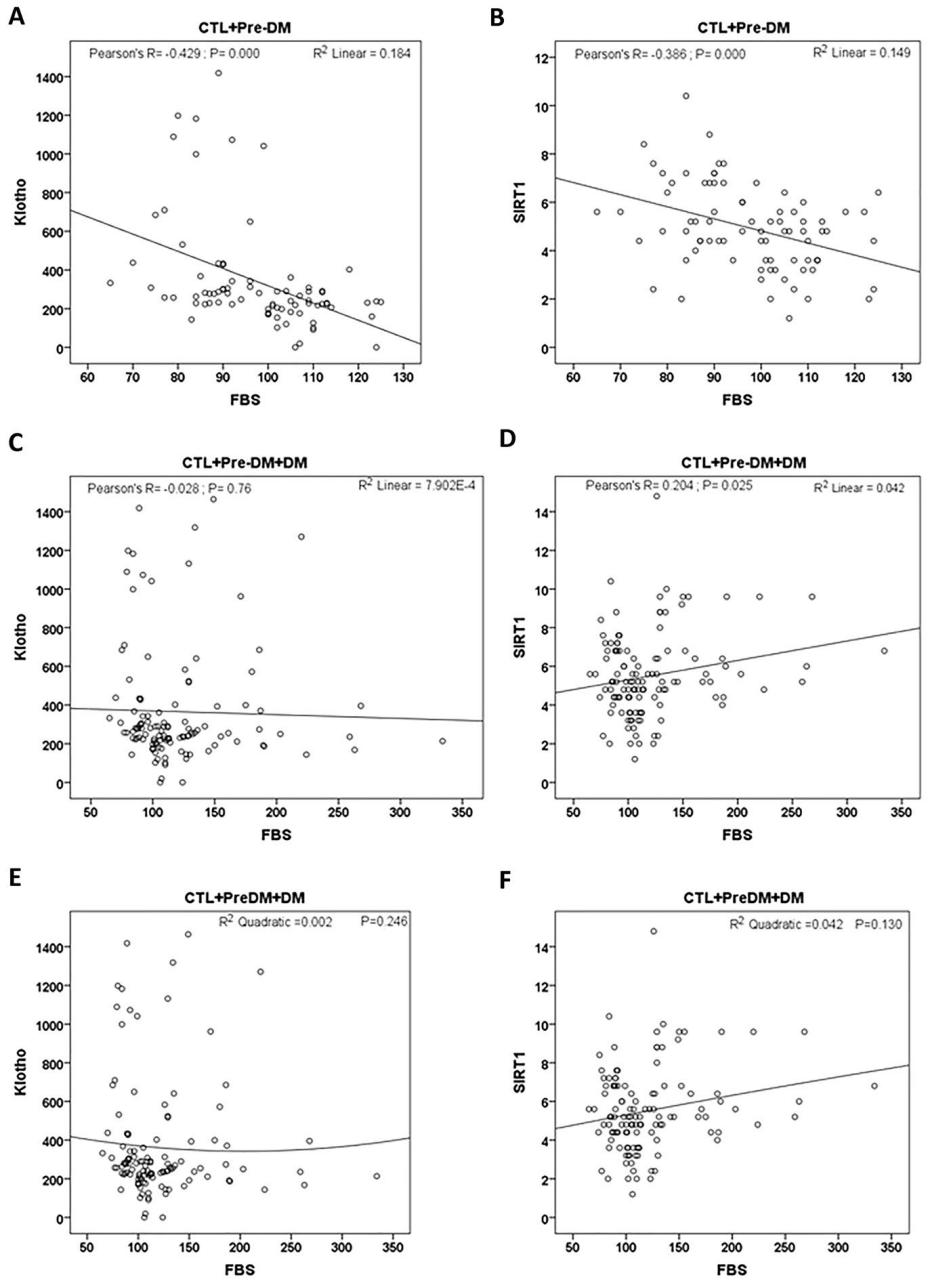
Correlations of serum levels of Klotho (**A**, **C**, **E**) or SIRT1 (**B**, **D**, **F**) with FBS over the normal/Pre-DM (**A**, **B**) or normal to DM range (**C**–**F**). **C**, **D** linear, and **E**, **F** quadratic versions. *CTL* control; *Pre-DM* pre-diabetes; *DM* diabetes mellitus; *FBS* fasting blood sugar

Correlation of serum Klotho and SIRT1 levels with FBS in studied groups are shown in Fig. 3. It seems that at the normal range of FBS the correlation of Klotho and SIRT1 is negative (Fig. 3A, B), but in higher FBS values the correlations reverses (Fig. 3C, D).

**Fig. 4:**
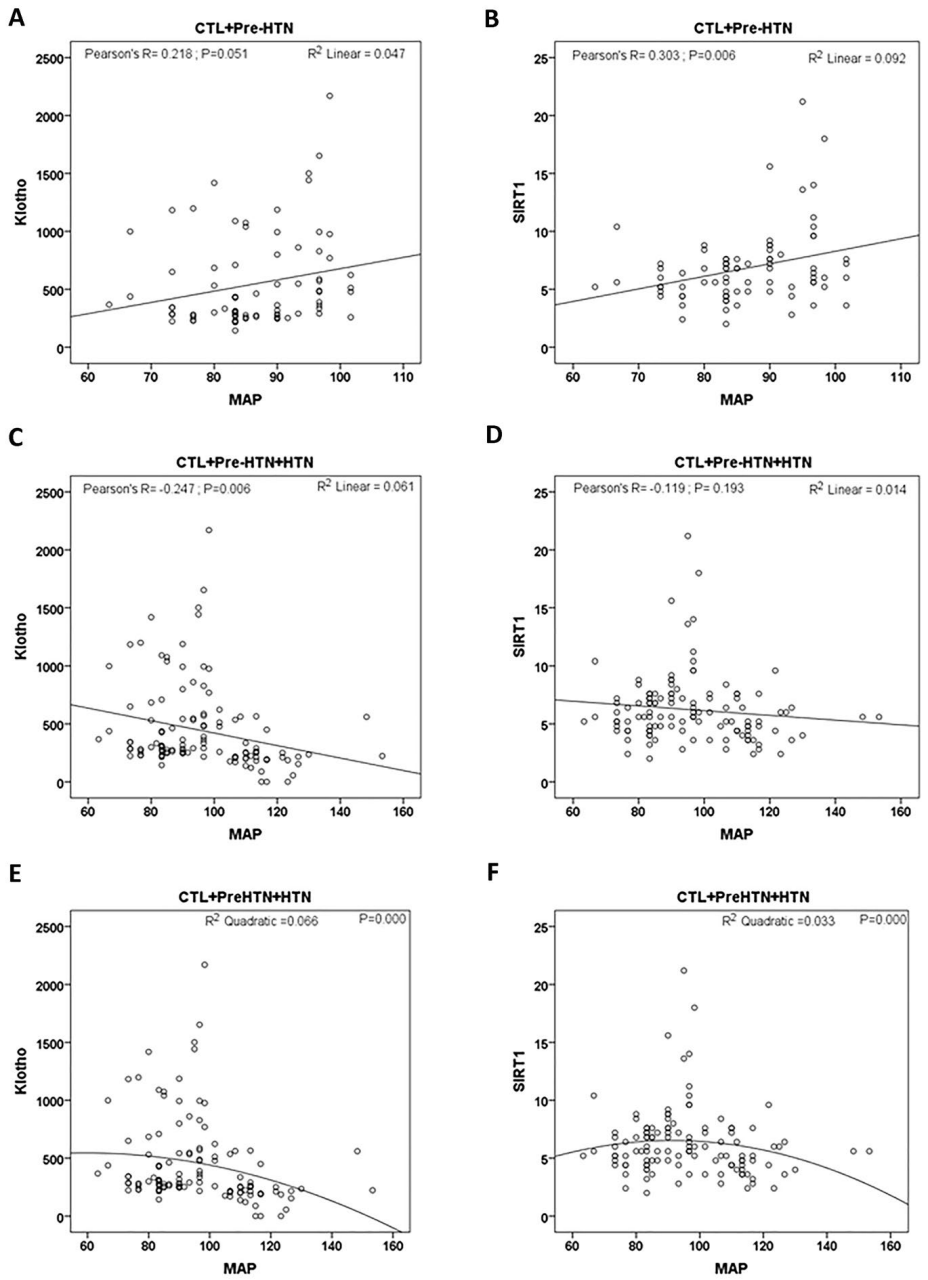
Correlations of serum levels of Klotho (**A**, **C**, **E**) or SIRT1 (**B**, **D**, **F**) with MAP over the normal/Pre-HTN (**A**, **B**) or normal to HTN (**C**–**F**). **C**, **D** linear, and **E**, **F** quadratic versions. *CTL* control; *Pre-HTN* pre-hypertension; *HTN* hypertension; *MAP* mean arterial pressure

Also we investigated the correlation of serum Klotho and SIRT1 levels with mean arterial pressure in the studied groups (Fig. 4). The correlation of the serum level of these two proteins with MAP at the normal range of BP is positive (Fig. 4A, B), but in higher MAP values the correlations reverses (Fig. 4C, D).

### Correlation between Klotho and SIRT1

Linear regression was performed to show the relationship between Klotho and SIRT1 themselves. The results showed that there was a significant relationship between them (P < 0.001, r = 0.601). Regression formula: SIRT1 (log10) = 0.435 + 0.016 × Klotho (square root).

### Predictors of serum Klotho and SIRT1 levels

Multiple linear regression was performed to determine the predictors of serum Klotho and SIRT1 levels. There was a significant negative correlation between serum Klotho level with age, BMI, FBS and MAP and a positive correlation with SIRT1 level. Serum SIRT1 levels was also positively correlated with serum Klotho level and only was negatively correlated with FBS (Table 2).

**Table 2:** Linear regression of serum Klotho or SIRT1 level, as dependent variables, with age, BMI, FBS, MAP in studied groups

Dependent variable	Predictor	USC Beta	SE	SC Beta	P value
Klotho	Age	− 0.072	0.031	− 0.123	0.019
	BMI	− 0.331	0.109	− 0.159	0.003
	FBS	− 0.037	0.008	− 0.246	0.000
	MAP	− 0.067	0.022	− 0.155	0.002
	Log of SIRT1	20.704	1.857	0.554	0.000
SIRT1	Age	0.000	0.001	0.025	0.663
	BMI	0.003	0.003	0.050	0.387
	FBS	0.001	0.000	0.143	0.012
	MAP	0.000	0.001	0.023	0.674
	SqR of Klotho	0.017	0.002	0.648	0.000

*USC Beta* unstandardized regression coefficient; *SC Beta* standardized coefficient; *SE* standard errors of the regression coefficients; *Klotho SqR* Klotho square root, *SIRT 1 Log* SIRT 1 logarithm, *FBS* fasting blood sugar; *BMI* body mass index; *MAP* mean arterial pressure

The comparison of Klotho level between the two genders of men and women showed that overall serum Klotho level was significantly higher in women than in men (406.9 ± 369.4 vs 322 ± 269.7 pg/ml) (P < 0.05), but serum SIRT1 level was not significantly different between the two genders.

## Discussion

We showed that serum levels of SIRT1 and Klotho decreased in pre-diabetic individuals and returned to normal in diabetic individuals. Serum Klotho and SIRT1 levels increase in pre-hypertensive people and decrease in hypertensive individuals. Linear regression also showed that changes in SIRT1 levels were positively related to changes in serum Klotho levels.

Serum levels of SIRT1 and Klotho in pre-diabetes were lower than in healthy controls and diabetics. Limited studies have examined the expression of SIRT1 and Klotho in pre-diabetes. In a study by Gateva et al. a non-significant lower serum Klotho level was observed in pre-diabetic obese people compared to normoglycemic obese people [20]. Also it was shown that SIRT1 expression in peripheral blood mononuclear cells [21] and abdominal fat [22] of pre-diabetic people decreases, which is in line with the results of the present study.

There are conflicting results about the expression of Klotho and SIRT1 in diabetic patients. Some studies have shown that serum Klotho levels in diabetic patients without nephropathy did not differ from healthy controls [23], which is similar to the results of the present study. However decrease [24] and increase [25] in serum Klotho levels has been also reported. There are also conflicting results about the expression of SIRT1 in diabetic patients [26, 27]. One of the reasons for these discrepancies can be due to the different stages of diabetes. It has been shown that one month after the onset of diabetes in rats, the expression of SIRT1 increases in the heart of animals, while three months later, the level of SIRT1 decreases [26]. The severity of diabetes in our study was not high as we did not include the patients who received treatment for diabetes in the study and HbA1C was about 7.5%, while in studies in which the level of Klotho and SIRT1 reduced, the severity of diabetes was higher [24, 28]. In addition as it was shown in Fig. 3A, B, in the physiologic range of FBS there is negative correlation between serum Klotho and SIRT1 with FBS. But when pathologic data ranges were added (Fig. 3C, D) the negative correlation was lost with Klotho and reversed to positive correlation with SIRT1. Collectively with other studies, in which it was shown that SIRT1 and Klotho levels decrease in advanced stages of diabetes, it seems that the correlation of these two proteins with FBS from prediabetes to early stage of the disease and finally to advanced stages has an inverted U shape pattern. This imply that the stage of the disease is a critical factor for determination of the levels of these two proteins in the serum.

It has been shown that insulin increases the concentration of Klotho by increasing the release and dissociation of Klotho from its membrane bound form [29]. At the onset of diabetes type 2 the pancreas is still able to produce insulin and the serum level of insulin is high in this conditions [30]. This leads to the release of Klotho from its membrane bound form and restore its serum levels to normal. However, as the disease progresses, the severity of nephropathy increases, and serum Klotho levels may decrease again [25].

SIRT1 and Klotho levels increase in pre-hypertensive patients before they decrease with the onset of hypertension and progression of the disease. As far as we know, no study has examined the serum levels of SIRT1 and Klotho in pre-hypertensive individuals. We observed that the level of Klotho decreases in hypertensive subjects which is in line with the findings of Su et al. [31] In addition, animal studies have shown that serum Klotho level in spontaneously hypertensive rats (SHR) is lower than in normotensive Wistar Kyoto (WKY) rats. Accordingly increasing the level of Klotho in SHR rats leads to lower blood pressure [32].

We showed that serum SIRT1 levels were lower in hypertensive group than control. In a study by Duman et al. the serum level of SIRT1 in hypertensive people was higher than healthy individuals [33]. In that study, only newly diagnosed hypertensive patients were examined, while we examined patients at higher stages of their disease. SIRT1 has been shown to increase in response to short-term stress while it reduces in long-term stresses [34]. Therefore the inverted U-shape of the relation between SIRT1 level and blood pressure found in the present study may be due to the longer duration of the disease in patients entered in our study. The level of Klotho and SIRT1 in patients with combined diabetes and hypertension is not different with those with pure hypertension. This may show that hypertension is a dominant determinant for the production of these regulatory proteins compared to diabetes.

Linear regression showed that there is a significant positive relationship between the serum level of Klotho and SIRT1. This is because they have common functions and may have common regulatory mechanisms. It seems that SIRT1 is downstream to the function of Klotho. It has been shown that SIRT1 level is reduced in Klotho deficient rats and activation of SIRT1 attenuates Klotho deficiency-induced arterial stiffness and hypertension [16]. The exact mechanism needs further investigation.

The results of multiple linear regression showed that there was a negative correlation between Klotho serum levels with age, BMI, FBS and blood pressure (Table 2). Given that these factors are age-dependent, and Klotho is an anti-aging protein that its level decrease with aging, this finding was expectable.

The reason for differential effect of gender on serum Klotho and SIRT1 found in the present study is unclear. Only in one study on acromegaly patients it was shown that serum Klotho levels were higher in women than in men. However after treatment by surgery the serum Klotho levels in these patients were still higher in women than in men [35]. This may be related to the positive effect of female sex hormones on the production of Klotho, a hypothesis that needs to be tested.

It seems that at the beginning of the diabetes and in pre-hypertension, the body increases the expression of these two proteins as a compensatory mechanism to counteract the unpleasant effects of the diseases on the body, but as the disease and its complications progresses, their expression decreases leading to overall U-shape relationship between their serum level with glucose level and a reverse U-shape relationship of them with the blood pressure level.

One of the limitations of our study was that according to our limited budget we tested the level of Klotho and Sirt1 in each sample once. However all serum samples were assessed by one Klotho or Sirt1 Kit, in a standard valid clinical laboratory with normal controls.

## Conclusions

There is a positive association between serum Klotho and SIRT1 levels. Also, the difference in the level of these two proteins in pre-diabetes with diabetes and in pre-hypertension with hypertension shows that the stage of the disease may play an important role in their expression. It seems that the responses of the body at the beginning of the diseases are compensatory but as the disease progresses, some adaptations are occurred.

## Data Availability

The datasets used and/or analysed during the current study are available from the corresponding author on reasonable request.

## Abbreviations

FBS: Fasting blood sugar
BP: Blood pressure
HbA1c: Hemoglobin A1c
SBP: Systolic blood pressure
DBP: Diastolic blood pressure
MAP: Mean arterial pressure
BMI: Body mass index
CTL: Control
Pre-DM: Pre-diabetes
DM: Diabetes mellitus
Pre-HTN: Pre-hypertension
HTN: Hypertension
Klotho SqR: Klotho square root
SIRT 1 Log: SIRT 1 logarithm
